# Rutting Behaviour of Geopolymer and Styrene Butadiene Styrene-Modified Asphalt Binder

**DOI:** 10.3390/polym14142780

**Published:** 2022-07-07

**Authors:** Abdulrahman Hamid, Hassan Baaj, Mohab El-Hakim

**Affiliations:** 1 Department of Civil and Environmental Engineering, University of Waterloo, Waterloo, ON N2L 3G1, Canada; a7hamid@uwaterloo.ca; 2 Department of Civil and Environmental Engineering, Manhattan College, Bronx, NY 10471, USA; mohab.elhakim@manhattan.edu

**Keywords:** asphalt binder, rheology, geopolymer, SBS, strain recovery, rutting

## Abstract

Modifying asphalt binders is an effective method of improving the performance of asphalt pavement, such as its resistance to rutting. However, because modification changes the behaviour of binders, substantial laboratory testing is required before field application to determine the best mixtures. This research aimed to evaluate the impacts of temperature, stresses, polymer type, and modification rate on the rutting behaviour of the asphalt binder modified with fly-ash-based geopolymer (GF), styrene butadiene styrene (SBS), and a combination of SBS and GF. The rheological properties of asphalt binders were investigated using the frequency sweep test at various temperatures. Additionally, the multiple stress creep recovery test was conducted at various temperatures and stresses to calculate the non-recoverable creep compliance (J${}_{nr}$) and the percent strain recovery (R). The rutting resistance of asphalt mixture was assessed using the Hamburg wheel rut test. The results revealed that the asphalt binder with 8% geopolymer (8%GF) exhibited the best response in terms of complex shear modulus (G${}^{*}$), rutting factor (G${}^{*}$/sinδ), R, and J${}_{nr}$ compared to the 4%GF and 12%GF at different temperatures. Another interesting finding is that GF’s use in the hybrid binder (2%SBS + 8%GF) led to a significant increase in the shear complex modulus and a decrease in the phase angle compared to the binder modified with 2%SBS. The geopolymer decreased the binder’s sensitivity to temperature for both unaged and RTFO asphalt binders. The hybrid binder would also improve strain recovery under high stress and temperatures and the ability to withstand severe traffic loads. Furthermore, there is a crucial relationship between temperature and J${}_{nr}$, which could help asphalt pavement designers select suitable modifiers considering the local climate and traffic volume.

## 1. Introduction

Rutting is a widely noticeable mode of distress, impacting the serviceability and quality of the roadway network. Rutting is a permanent deformation that occurs in the direction of traffic due to the accumulation of unrecoverable strain from repeated loads applied to the asphalt pavement [1]. The existing SHRP binder specifications, such as PG performance, fatigue parameter, and rutting parameter, were determined based on the behaviour of the asphalt binder in the linear viscoelastic range. In contrast, the asphalt binder in the mixture has a nonlinear response under high stress and strain conditions [2]. Therefore, these specifications cannot exactly capture or correlate the asphalt binder’s performance in the mixture.

Polymer modification of asphalt binders has been considered in recent decades as a significant method for improving rutting performance and pavement durability [3,4,5]. Asphalt binder modification is a valuable approach for improving the viscoelastic properties of asphalt binders. However, it introduces further complexity to the behaviour of asphalt binders. The MSCR test was designed to obtain the nonlinear response of the asphalt binder and link that response to rutting in the asphalt mixture [2]. The multiple stress creep-recovery (MSCR) test has been widely utilised to predict the influence of polymer-modified asphalt binders on creep recovery [6,7,8,9]. The MSCR test was also efficiently conducted and designed to be a sign of rutting performance in the field of modified and unmodified asphalt binders [10]. The MSCR test essentially uses a sequence of creep and recovery cycles at various stress levels. The concept is that when shear stress is removed, the viscoelastic strain generated in the creep part can be recovered, allowing the permanent strain to be separated from the total strain, which could be used to predict field rutting [11].

There has been a lot of effort put toward establishing a link between the MSCR test findings and the rutting performance of asphalt mixtures as assessed by field studies [2,12] or laboratory studies [2,13,14,15,16,17,18]. It has been reported that the non-recoverable creep compliance (J${}_{nr}$) has a better relationship with the asphalt mixture rutting depth using different asphalt mixture rutting tests, such as the Hamburg wheel tracking test (HWTT) [2,13,17], and the wheel tracking test [14,15,18]. Behnood et al. [16] conducted a laboratory investigation using the flow number test to correlate the flow number at 51 ${}^{\circ }$C with the asphalt binder rutting parameter G${}^{*}$/sinδ and J${}_{nr}$ at 64 ${}^{\circ }$C and different stresses (0.1 kPa and 3.2 kPa). The results showed that the best correlation between flow number and J${}_{nr}$ occurred at 3.2 kPa. Furthermore, the MSCR parameter (J${}_{nr}$) correlates better with mixture rutting than the G${}^{*}$/sinδ parameter [2,10]. Al-Adham et al. [19] concluded that J${}_{nr}$ and R have statistically significant relationships with mixture rutting.

Recently, there has been increasing awareness about decreasing the use of asphalt binders and reducing energy used during the preparation of asphalt concrete. Consequently, the need for new additives with properties leading to enhancement of the asphalt binder properties is constantly growing. Moreover, reused waste materials would reduce the rehabilitation and storage costs of these materials and would provide a financial benefit to the producers. The term ’geopolymer’ was first formulated by Davidovits [20], which can be produced using pozzolanic materials, such as fly ash, metakaolin, and slag, with alkaline solutions, such as sodium hydroxide (NaOH) and sodium silicate (Na${}_{2}$SiO${}_{3}$) or potassium hydroxide (KOH) and potassium silicate (K${}_{2}$SiO${}_{3}$) [21]. The geopolymer is an eco-friendly material that is produced using little energy and releases small amounts greenhouse gas emissions during its manufacture. Geopolymers can be made from materials that comprise reactive or amorphous silica and alumina [22,23].

Thus, using a geopolymer as a modifier can significantly impact the amount of the asphalt binder used, which will decrease the CO${}_{2}$ emissions during the asphalt binder’s production. Hamid et al. [24] used fly ash and glass powder-based geopolymer as a modifier for asphalt binder at different percentages. The geopolymer-modified asphalt binder increased the fatigue resistance compared to the virgin binder. Hamid et al. [25] noted that a fly-ash-based geopolymer had an insignificant impact on the microstructure of the asphalt binder. Tang et al. [26] used metakaolin, slag, and silica fume as alumino-silicate precursors and sodium hydroxide and sodium silicate as activators to make the geopolymer as a warm mix asphalt (WMA) additive. Using a geopolymer as an additive resulted in a 50% cost savings when compared to zeolite additives.

Moreover, geopolymers are used to improve the properties of recycled pavement materials. Hoy et al. [27] utilised fly ash (FA) and fly-ash-based geopolymer with recycled asphalt pavement (RAP) and investigated the unconfined compressive strength (UCS) for RAP-FA mix and RAP-FA geopolymer. The results showed that the UCS of RAP-FA geopolymer is greater than the UCS of RAP-FA mix; the UCS of RAP-FA geopolymer is affected by the NaOH/Na${}_{2}$SiO${}_{3}$ ratio. Decreasing the NaOH/Na${}_{2}$SiO${}_{3}$ ratio showed a substantial increase in the UCS. Moreover, this investigation confirmed that RAP-FA blends and RAP-FA geopolymers could be utilised as stabilisers for pavement materials. Arulrajah et al. [28] used calcium carbide residue, fly ash, and slag as an aluminosilicate resource, with sodium silicate and sodium hydroxide as activators to produce the geopolymers. The study aimed to stabilise the recycled concrete aggregates (RCA) and crushed brick (CB) using a geopolymer. The results indicated that modified RCA and CB could be used as base and subbase materials, and the resilient modulus of modified CB improved significantly.

There is a global movement toward lowering pollution through reducing by-products and waste materials. Therefore, governments set aside a significant budget to develop ways to incorporate these materials with the raw materials used in many fields. The amount of fly ash has increased worldwide because of increasing human activity, which has resulted in more landfill space being utilised to dispose of these materials. Thus, utilising these materials as aluminosilicate sources during the geopolymer production to enhance the asphalt binder’s properties and to decrease the use of asphalt binder during the construction of flexible pavement, would have significant economic and environmental benefits. Therefore, this study aimed to investigate the feasibility of using the geopolymer as a modifier to enhance the rutting resistance of asphalt binder by investigating the temperature and modification rate effects on the rheological behaviour of asphalt binder; the influence of geopolymer on the percent creep recovery (R) and non-recoverable creep compliance (J${}_{nr}$) of asphalt binder; the effect of modifiers on traffic loading at different temperatures; and the rutting resistance of asphalt concrete with different additive types.

### Chemical Interactions in Geopolymer

The geopolymer is formed when the aluminosilicate source, such as fly ash, reacts with the alkaline solution. This reaction can be summarised into the following steps [29]: (a) Hydroxyl ions (OH${}^{-}$) in the highly concentrated alkaline solution cause the dissolution of fly ash minerals, such as alumina and silica. (b) Diffusion of the silica and alumina monomers, which interact to form dimers, trimers, tetramers, and so on. (c) Condensation with sodium cations (Na${}^{+}$) to form the N-A-S-H gel with time. This gel changes with time [30], whereby the initial gel 1 consists of high amounts of alumina ions in the early stages of the reaction because the Al–O bond is weaker than the Si–O bond. Consequently, gel 2 is formed due to increasing the Si–O bond number with time, which raises the silicon concentration in the N-A-S-H gel. (d) The last step is crystallisation to hardening, whereby the tetrahedral silica (SiO${}_{4}$) and alumina (AlO${}_{4}$) are joined by oxygen (O${}_{2}$) in the three-dimensional chain networks that are called geopolymers.

## 2. Methodology

### 2.1. Materials Preparation

#### 2.1.1. Geopolymer Preparation

The geopolymer was made up of an alkali activator and a fly ash mixture. Alkali activators included Na${}_{2}$SiO${}_{3}$ and NaOH at a concentration of 8 molar. Before making the geopolymer, the NaOH solution was made in a fume cabinet by dissolving NaOH in deionised water for one day. Table 1 presents the chemical composition of fly ash (Class F). The low-calcium fly ash (Class F) used in this study contained more silica and alumina than the high calcium fly ash (Class C), as recommended by other researchers [31,32,33]. It was shown that the curing time and type of activator have substantial influences on chemical reactions during the preparation of geopolymer [30]. Additionally, it is noted that the sodium hydroxide and sodium silicate combination provides a geopolymer with high mechanical performance [34]. The maximum yield stress and storage modulus values are obtained when 8 moles of NaOH is used as an activator during the preparation of the fly-ash-based geopolymer [35]. The alumino-silicate precursors in fly ash were activated using a 1:2 mass ratio of Na${}_{2}$SiO${}_{3}$ and NaOH solution, with 200 g of fly ash blended with 80 g of the alkaline medium for 5 min. The resulting slurry was poured into the silicon molds. The geopolymers were then cured for six days at room temperature (23–25 ${}^{\circ }$C) before being cured for 24 h at 65 ${}^{\circ }$C. Finally, geopolymer samples were ground into powder and sieved utilising sieve number 100 to remove particles larger than 0.15 mm in diameter, since the MSCR test does not apply to an asphalt binder with particles larger than 0.25 mm [36].

#### 2.1.2. Asphalt Binder Preparation

The modified asphalt binders were made using two different methods in this investigation. In the first method, the geopolymer-modified asphalt binder was prepared by heating 300 g of asphalt binder (PG 58-28) at 140 ${}^{\circ }$C. The geopolymer was then mixed into the neat asphalt binder in various proportions. To prepare a homogeneous blend, a mechanical shear mixer was employed at a speed of 2000 r/min for 60 min while maintaining a temperature of 140 ${}^{\circ }$C ± 5. Hamid et al. [25] concluded that changing asphalt binder with varying percentages of geopolymer (3, 6, and 9%) has no effect on the microstructure of the asphalt binder.

In the second method, the SBS modified asphalt binder was prepared by heating the asphalt binder (PG 58-28) to 170 ${}^{\circ }$C. The SBS polymer has a linear microstructure with 31.6% styrene content. The SBS was blended with asphalt binder using the high shear mixer and heating mantle at a speed of 2000 r/min and a temperature of 170 ${}^{\circ }$C ± 5 for 60 min. At the end of the hour, 10% of the crosslinking agent was added and mixed in for 30 min. Finally, a curing time of another 60 min was employed while controlling the temperature at 180 ${}^{\circ }$C ± 5 and reducing the high shear mixer speed to 1000 r/min. Following the mixing process, samples for short-term ageing in a Rolling Thin Film Oven (RTFO) were prepared according to AASHTO T 240 [37]. Then, all RTFO specimens were subjected to the frequency sweep and MSCR tests using the Dynamic Shear Rheometer (DSR). Table 2 shows the asphalt binder modification process. Table 3 presents the sieve analysis of the aggregates used to produce HMA mixtures using the neat and modified asphalt binders.

### 2.2. Experimental Procedures

#### 2.2.1. Rotational Viscosity

The neat and modified asphalt binders’ viscosities were determined at 135 and 165 ${}^{\circ }$C according to AASHTO T 316 [38]. Each test result was calculated using the average of three readings for each temperature.

#### 2.2.2. Dynamic Shear Rheometer (DSR)

The DSR was applied to evaluate neat and modified binders’ rheological behaviour, according to AASHTO T 315 [39]. A frequency sweep test was performed to study the influences of loading frequency, temperature, and additives on the asphalt binders’ rheological properties. Two samples for each binder were tested, and the average was identified as the test result. Different frequencies ranging from 0.0159 to 15 H${}_{z}$ were applied to different test temperatures (40, 46, 52, 58, 64, and 70 ${}^{\circ }$C).

**Figure 1:**
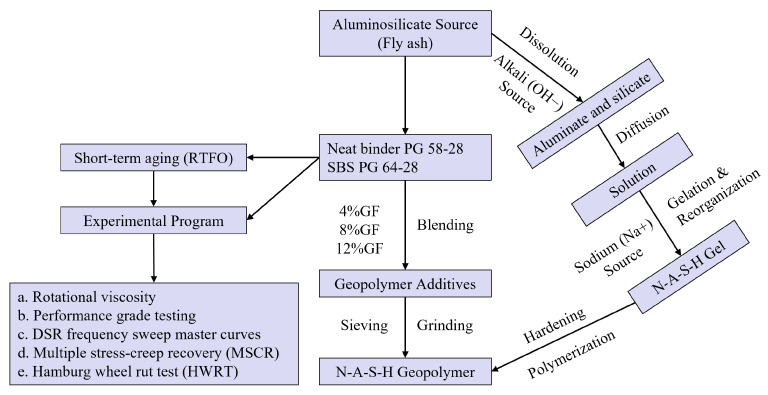
Research methodology.

The MSCR test was performed on asphalt binders using 25 mm diameter samples in accordance with AASHTO T350 [40] to determine percent recovery (R) and non-recoverable creep compliance (J${}_{nr}$). All asphalt binder samples were tested in creep and recovery at low and high stress levels of 0.1 and 3.2 kPa. The average of two repeated samples was used to calculate the test result for each temperature. The test was carried out using a creep time of 1 s and a recovery time of 9 s. Figure 1 summarises the research methodology.

#### 2.2.3. Hamburg Wheel Rut Test

Rutting is a common sign of distress that affects the road network’s serviceability and quality. Rutting is a permanent deformation that appears in the traffic direction because of unrecoverable strain accumulated by repetitive loads applied to the asphalt pavement [1]. The Hamburg wheel rut test (HWRT) was developed to investigate the resistance of asphalt mixtures to rutting and moisture damage. The tests were conducted in accordance with AASHTO T 324 [41]. The Superpave Gyratory Compactor was used to prepare four samples 150 mm in diameter and 63 mm in height at 7% ± 0.5 air. The asphalt mixture samples were submerged and conditioned for 30 min at 44 ${}^{\circ }$C in the water bath. Solid steel wheels were used to test the samples, and linear variable differential transformers (LVDTs) were applied to determine the average rutting depth.

## 3. Results and Discussion

### 3.1. Effects of Additives on Rheological Properties

#### 3.1.1. Viscosity Results

**Figure 2:**
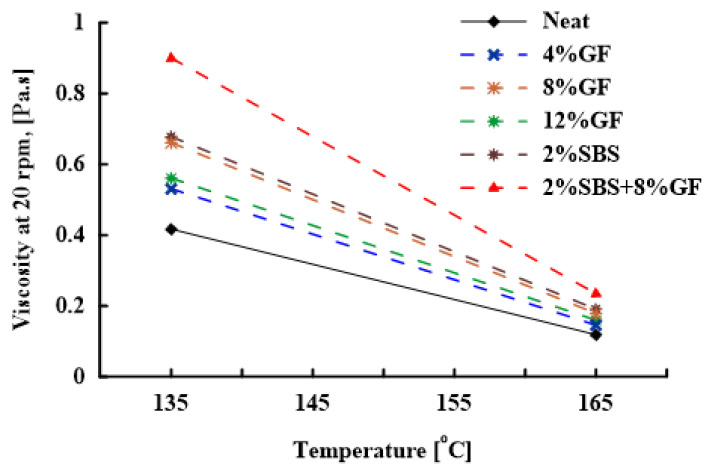
Rotational viscosity of neat and modified asphalt binders.

The viscosity of asphalt mixtures is a significant factor in establishing the temperature range for mixing and compaction. Therefore, the viscosities of neat and modified asphalt binders were measured at 135 and 165 ${}^{\circ }$C to investigate the additives’ influences on the viscosity. Figure 2 shows that the viscosity of the asphalt binder increases as the modifier percentage increases. Increasing viscosity is attributed to the larger particles present in the fluid [42], which could be induced by the formation of chain networks in the asphalt–geopolymer mixture. The viscosity of the SBS-modified asphalt binder was significantly greater than that of the asphalt binders modified with a geopolymer (4%, 8%, and 12%). A T-test was performed to examine the statistical significance between the viscosity of neat versus SBS-modified and geopolymer-modified binders. The *p*-values were 1.09 × 10${}^{-4}$ and 1.24 × 10${}^{-4}$, respectively. The hybrid binder (2%SBS + 8%GF) had the highest viscosity compared to neat and other modified asphalt binders.

A two-way analysis of variance (ANOVA) was performed to examine the significance of the variations in viscosity resulting from the geopolymer and SBS modifications to the binders. The ANOVA test was performed using the data at 135 and 165 ${}^{\circ }$C. The ANOVA test results indicated that binder modifications had a considerable strong impact on viscosity (*p*-value = 1.9 × 10${}^{-151}$), and the temperature had a strong influence on viscosity results as well (*p*-value = 2.4 × 10${}^{-154}$). Results obtained from the ANOVA test are presented in Table 4. A statistical t-test was performed to examine the significance of the variation in viscosity between the hybrid and neat binders at 135 and 165 ${}^{\circ }$C. The viscosity of the hybrid binder was 116% (*p*-value = 9.2 × 10${}^{-24}$) and 97% (*p*-value = 1.56 × 10${}^{-15}$) higher than the neat at 135 and 165 ${}^{\circ }$C, respectively. Consequently, the hybrid asphalt binder demonstrated high viscosity compared to the other modified asphalt binders. The results also revealed a minor influence of geopolymer content on the viscosities of 4%GF and 12%GF binders at 135 ${}^{\circ }$C.

#### 3.1.2. Temperature’s Effects on the Complex Shear Modulus and Phase Angle

**Figure 3:**
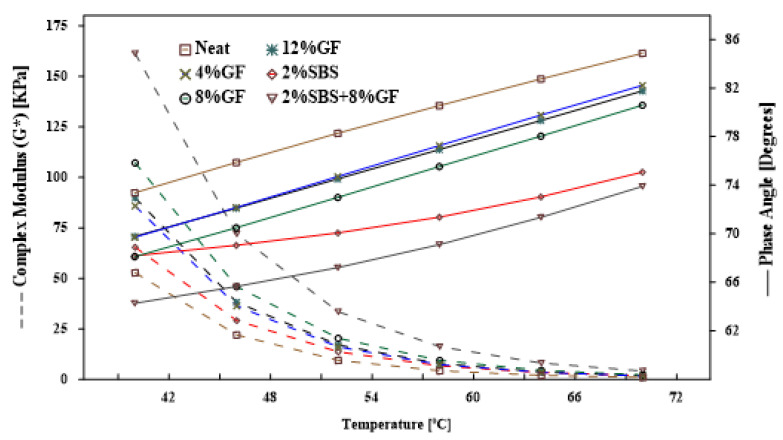
Temperature effect on the complex shear modulus and phase angle at 10 rad/s.

Variations in complex shear modulus and phase angle for neat and modified asphalt binders among different testing temperatures are shown in Figure 3. Throughout the test temperature range, the complex modulus of the geopolymer-modified asphalt binder was increased by adding up to 8% more geopolymer. However, beyond 8% GF, the shear complex modulus of the asphalt binder dropped. That trend was quite similar to the one observed for viscosity. A closer look at the results of the shear complex modulus of geopolymer-modified asphalt binder with 4% and 12% revealed that the difference between the two binders was statistically insignificant (*p*-value = 0.44). Table 5 demonstrates the importance of the difference in shear modulus of several modified asphalt binders compared to the neat binder at 10 rad/s (1.59 H${}_{z}$) using multiple F-tests. The SBS-modified asphalt binder had a lower shear complex modulus and phase angle than the GF-modified asphalt binders throughout the test temperature range. SBS is a thermoplastic elastomer material which is composed of a short chain of polystyrene, a long chain of polybutadiene, and finally, another short chain of polystyrene. The polybutadiene blocks are crosslinked by the polystyrene clusters. The polystyrene clusters break and produce a short chain when SBS is heated. The modulus then starts to drop. On the other hand, the SBS utilised in this work included approximately 70% butadiene, which gave the asphalt binder its elastic behaviour, and this explains the drop in phase angle value.

In contrast, the hybrid asphalt binder exhibited complex shear modulus values almost double (up to 222% increase) those achieved by adding 2% of SBS to the asphalt binder, and it had the smallest phase angle compared to all the other asphalt binder modifications. For example, the shear complex modulus increased from 22 to 72 kPa, and the phase angle dropped from 74.5 to 65${}^{\circ }$ at 46 ${}^{\circ }$C compared to the neat binder. The beneficial interaction between GF and SBS, and the effectiveness of the geopolymer modification rate (4%, 8%, and 12%), could explain this. This reduction in the phase angle indicates the change in the viscoelastic behaviour and a shift toward elastic behaviour. However, the results of phase angles cannot be used to figure out which asphalt binder type is more elastic. Therefore, the storage and loss modulus of the complex shear modulus should be separated.

**Figure 4:**
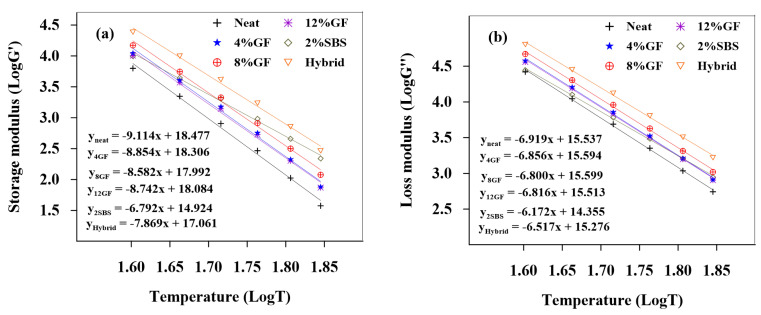
Temperature sensitivity of the (**a**) storage and (**b**) loss modulus of unaged binders.

**Figure 5:**
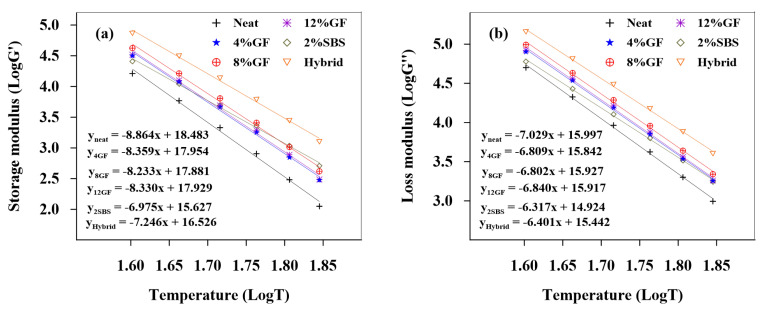
Temperature sensitivity of the (**a**) storage and (**b**) loss modulus of RTFO binders.

Figure 4 and Figure 5 show the logarithms of storage modulus (G${}^{\prime }$) and loss modulus (G${}^{\prime \prime }$) versus temperatures for unaged and RTFO binders, respectively. The equations for linear regression were created and are depicted in the figures. Table 6 displays the absolute values of the linear regression equations’ slopes. The slopes depict the asphalt binders’ temperature sensitivity; the steeper the slope, the more sensitive the asphalt binder is to temperature changes.

The results showed that the modifiers have significant impacts on the binder’s sensitivity to temperature. The temperature sensitivity of G${}^{\prime }$ and G${}^{\prime \prime }$ for both unaged and RTFO modified asphalt binders decreased. The results indicate that the modifiers had a significant impact on the binder’s sensitivity to temperature, whereby the temperature sensitivity of G${}^{\prime }$ and G${}^{\prime \prime }$ for both unaged and RTFO modified asphalt binders decreased.

#### 3.1.3. Temperature Effect on the Rutting Factor

**Figure 6:**
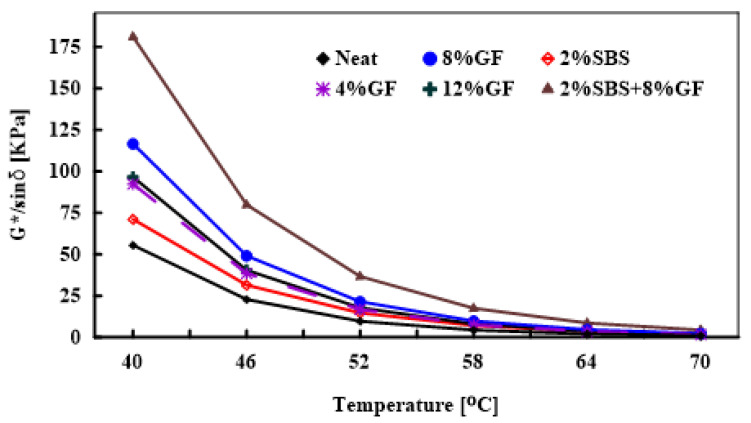
Temperature’s effect on the rutting factors of neat and modified asphalt binders at 10 rad/s.

The impact of the temperature on the rutting factors (G${}^{*}$/sinδ) of neat and modified asphalt binders is presented in Figure 6 and Table 7. To achieve rutting standards, the rutting factor for an unaged asphalt binder must be at least 1 kPa, and it must be at least 2.2 kPa for an aged asphalt binder at 10 rad/s, according to the Superpave specifications [1]. The data were analysed using the ANOVA statistical test to examine the effects of temperature and asphalt binder modification on the G${}^{*}$/sinδ. Table 8 presents the ANOVA test results of temperature and binder modification. The results indicate a statistically considerable impact on G${}^{*}$/sinδ with *p*-values of 4.33 × 10${}^{-60}$ and 9.88 × 10${}^{-44}$ for temperature and asphalt binder modification percentage, respectively. The results show that increasing the polymer additives increased the rutting factor, indicating an increase in rutting resistance. These results were also noted in previous research [9,43,44]. Multiple t-tests were conducted to specify the significance of asphalt binder modification on G${}^{*}$/sinδ. For example, the rutting factor of the 8%GF-modified asphalt binder at 58 ${}^{\circ }$C was 124% higher than that of the neat asphalt binder. However, the difference was statistically insignificant with a *p*-value = 0.19. While the rutting factor of 2%SBS increased only 64% more than the neat asphalt binder, the difference was statistically insignificant (*p*-value = 0.54).

The combination of SBS and geopolymer (2%SBS + 8%GF)-modified asphalt binder produced an increase in the rutting factor that reached up to 300% more than the neat binder at 58 ${}^{\circ }$C with a *p*-value = 0.059, as summarised in Table 7. Consequently, the hybrid asphalt binder remained superior in rutting resistance potential compared to other modified asphalt binders. This could be attributed to the geopolymer modification rate and the positive interaction between SBS and the geopolymer.

#### 3.1.4. Frequency’s Effect on the Rutting Factor

**Figure 7:**
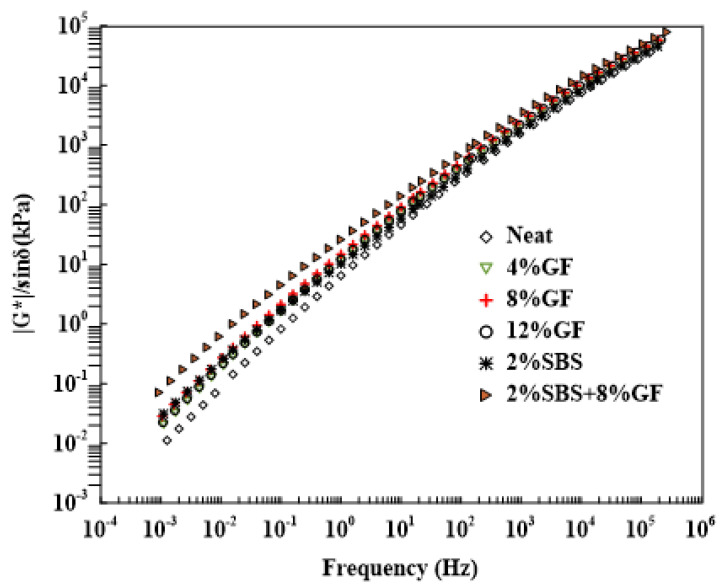
Temperature’s effect on the rutting factors of neat and modified asphalt binders at 10 rad/s.

The master curve of rutting factor was generated for the neat and various modified asphalt binders at 52 ${}^{\circ }$C reference temperature, as shown in Figure 7. The frequency sweep test was carried out at high temperatures (46, 52, 58, 64, and 70 ${}^{\circ }$C) with varied frequencies ranging from 0.0159 H${}_{z}$ to 15 H${}_{z}$. At high temperatures, higher |G${}^{*}$|/sinδ values are preferred to reduce the energy dissipation due to repeated loading. The less energy dissipated per cycle, the higher the rutting-resistance of the asphalt mixture [9]. The modified asphalt binders achieved the highest rutting resistance compared to the neat asphalt binder. It should be noted that the hybrid asphalt binder had the strongest potential for rutting resistance over a wide range of loading frequencies, whereby the combination of SBS and geopolymer increased the asphalt binder’s stiffness. Meanwhile, the 8%GF had a somewhat similar impact on the rutting resistance at low frequencies while having a higher rutting parameter than the 2%SBS version at high frequencies.

The impact of various frequencies on the ∣G${}^{*}$∣/sinδ of neat and modified asphalt binders at 52 ${}^{\circ }$C is summarised in Table 9. At low modified frequency (10${}^{-2}$ H${}_{z}$), the rutting factor of 4%GF, 8%GF, and 12%GF binders increased by 184%, 286%, and 214%, respectively, compared to the neat asphalt binder. While the 2%SBS binder showed a rutting factor almost the same as achieved by adding 8% of GF to the asphalt binder, whereas the combination of 2%SBS and 8%GF attained an increase in 786% compared to the rutting factor of the neat asphalt binder. The ANOVA was applied to test the significance of asphalt binder modification and loading frequency on the ∣G* ∣/sinδ.

Table 10 presents the results of the ANOVA test. Loading frequency has a statistically significant impact on ∣G${}^{*}$∣/sinδ, with a *p*-value of 5.61 × 10${}^{-22}$. The effect of asphalt binder modification on ∣G${}^{*}$∣/sinδ is also statistically significant with a *p*-value of 5.45 × 10${}^{-15}$. At a high modified frequency (10 H${}_{z}$), the rutting factor of 2%SBS increased up to 30%, which showed the lowest rutting factor. While the hybrid asphalt binder also indicated the highest rutting factor with 207% more than the neat asphalt binder, as reported in Table 9. This confirms that the SBS and geopolymer combination has a considerable rutting performance improvement over the other modifiers.

### 3.2. Analysis Multiple Stress Creep-Recovery Test Results

#### 3.2.1. Effects of Additives on the Creep Recovery Behaviour

**Figure 8:**
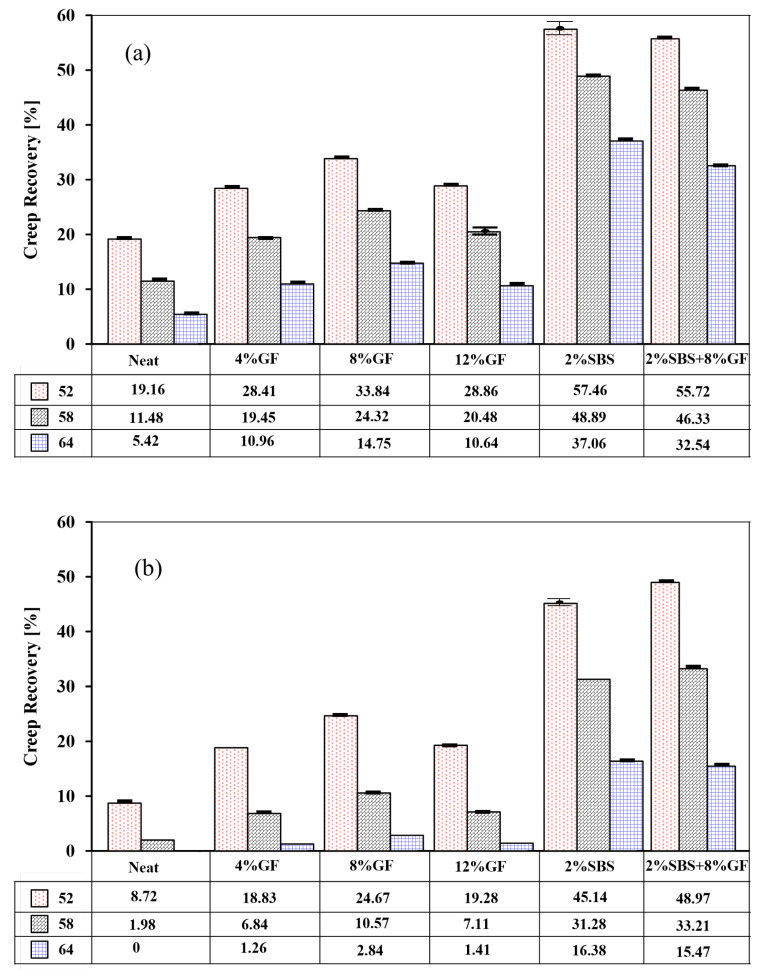
Creep recovery of neat and modified asphalt binders at (**a**) 0.1 kPa and (**b**) 3.2 kPa.

The MSCR test was carried out under two stress levels (0.1 kPa and 3.2 kPa) and a wide range of temperatures from 46 to 70 ${}^{\circ }$C with an increment of 3 ${}^{\circ }$C. The figures included in this paper present the average results obtained from three temperatures (52 ${}^{\circ }$C, 58 ${}^{\circ }$C, and 64 ${}^{\circ }$C) due to page-space limitation. However, the statistical analysis of the data was performed on the data obtained from all testing temperatures. The influence of temperature, stresses, and polymer types on the recovery and non-recovery of asphalt binders was investigated. Percent-strain creep recovery, for neat and modified asphalt binders, at various temperatures (52, 58, and 64 ${}^{\circ }$C) for low (0.1 kPa) and high (3.2 kPa) stress levels are shown in Figure 8a,b, respectively. The efficiency of geopolymer and SBS modification rates is reflected at all stress levels during all test temperatures. Additionally, the 8%GF binder showed the highest creep recovery compared to 4%GF (*p*-value = 2.5 × 10${}^{-3}$ and *p*-value = 3.34 × 10${}^{-6}$ for 0.1 kPa and 3.2 kPa, respectively) and 12%GF (*p*-value = 1.46 × 10${}^{-3}$ and *p*-value = 4.38 × 10${}^{-6}$ for 0.1 kPa and 3.2 kPa, respectively).

The recoverable strain increased by 23% and 32% by increasing the geopolymer percentage from 4% to 8% at 0.1 kPa and 3.2 kPa, respectively. The recoverable strain decreased by 15% and 20% by increasing the geopolymer percentage from 8% to 12% at 0.1 kPa and 3.2 kPa, respectively. However, the creep recovery of the 2% SBS binder was higher than that of the 8%GF binder at all temperatures and stress levels (*p*-value = 9.77 × 10${}^{-18}$ and 6.73 × 10${}^{-10}$ for 0.1 kPa and 3.2 kPa, respectively). Comparing 2%SBS and hybrid binders, it can be noted that 2%SBS binder showed a slight insignificant increase in 5% in creep recovery at 0.1 kPa of stress (*p*-value = 0.64) as shown in Figure 8a. While the hybrid asphalt binder performed at 6% insignificant higher creep recovery at 3.2 kPa (*p*-value = 0.77) as shown in Figure 8b. This is attributed to the various interaction mechanisms within the asphalt binder matrix for each modifier, which needs closer analysis of every modifier’s physical and chemical interactions within the asphalt binder and their potential influences on the microstructure of the asphalt binder.

**Figure 9:**
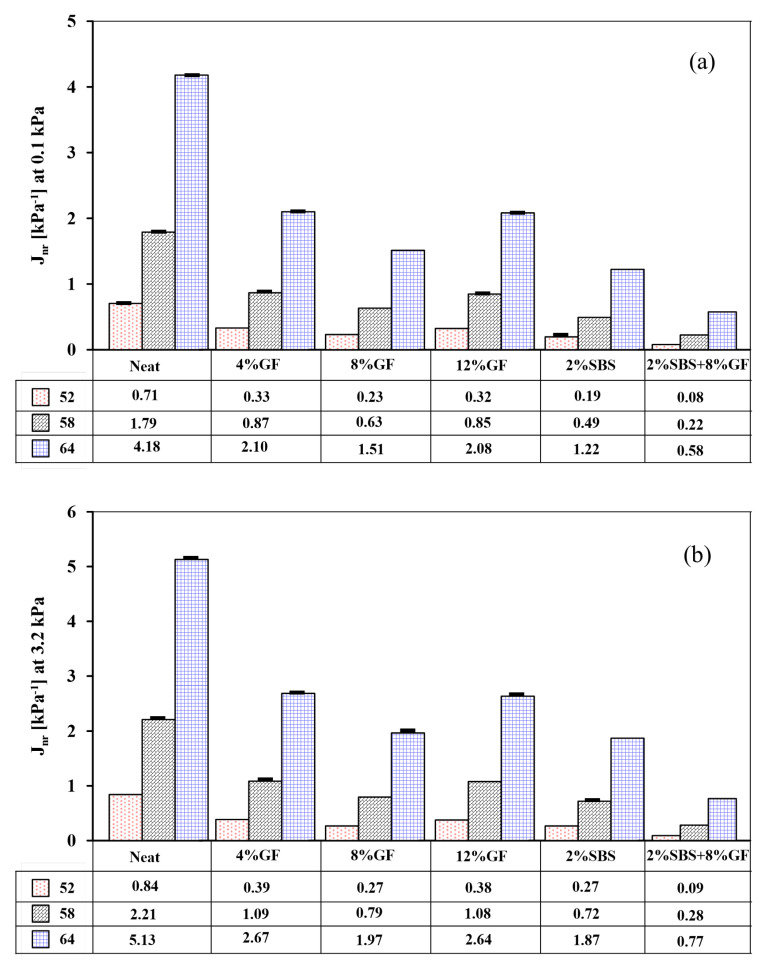
Non-recoverable creep compliance of neat and modified asphalt binders at (**a**) 0.1 kPa and (**b**) 3.2 kPa.

Figure 9a,b presents the non-recoverable creep compliance of the neat and modified asphalt binders at 0.1 kPa and 3.2 kPa stresses, respectively. Generally, non-recoverable creep compliance with a low value is needed for acceptable rutting resistance. Simultaneously, asphalt binder with 0.5 kPa${}^{-1}$ or less of J${}_{nr}$ at 3.2 kPa is suitable for extremely heavy traffic (≥30 million ESALs), according to the AASHTO standard [36]. The results revealed that the hybrid binder significantly outperformed all modifiers in terms of non-recoverable creep compliance at 0.1 kPa and 3.2 kPa.

Comparing the hybrid binder to the binder with the closest non-recoverable creep compliance strain, which was 2%SBS, the hybrid binder performed at 54% and 59% lower non-recoverable creep strain with *p*-values of 6.5 × 10${}^{-4}$ and 8.7 × 10${}^{-4}$ at 0.1 and 3.2 kPa, respectively. In addition, it can be observed that the 2%SBS binder showed a 17% reduction in non-recoverable strain values compared to the 8%GF at the 0.1 kPa stress level (*p*-value = 6.4 × 10${}^{-4}$) and an insignificant difference of 2% in the non-recoverable strain at the 3.2 kPa stress level (*p*-value = 0.05). These observations are essential, considering that the 2%SBS binder showed similar strain recovery to the hybrid binder at 3.2 kPa, for various temperatures, as shown in Figure 8. This reconfirms that the hybrid modification with GF and SBS led to superior strain recovery compared to the GF or SBS alone.

**Figure 10:**
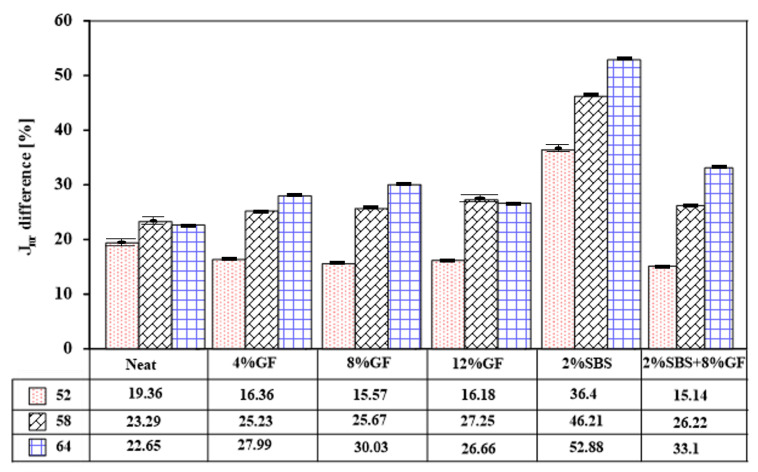
Non-recoverable creep compliance difference between neat and modified asphalt binders.

Figure 10 presents the difference in non-recoverable creep compliance strain (J${}_{nr}$), which was calculated as a percent increase in J${}_{nr}$ between 0.1 and 3.2 kPa stress levels. This (J${}_{nrdiff}$) factor is an indicator of the stress sensitivity of modified asphalt binders. All modified asphalt binders showed J${}_{nrdiff}$ below the maximum limit (75%) that was recommended by the AASHTO standard [36]. However, it appears that the SBS-modified binder has high sensitivity to stress levels compared to the hybrid and GF-modified binders at different temperatures.

#### 3.2.2. Influences of Temperature and Polymer Types on the Traffic Level

**Figure 11:**
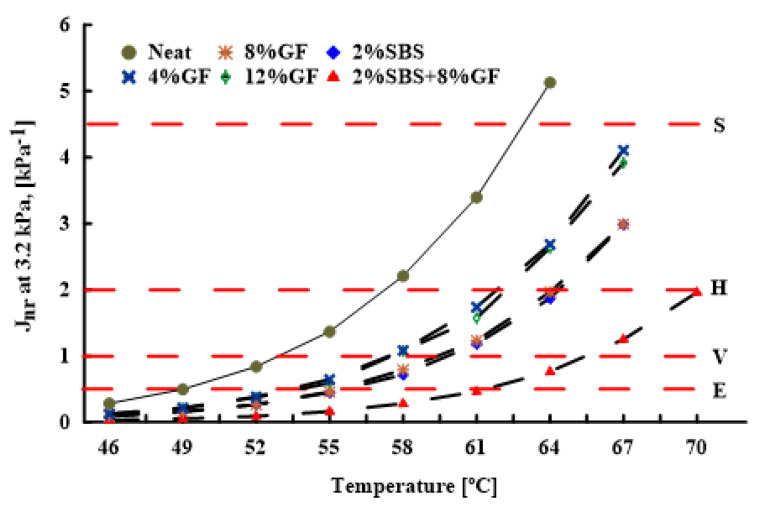
Temperature and additives effects on the traffic level.

MSCR test results are influenced by temperature and polymer types and amounts. Figure 11 illustrates the impacts of temperature and polymer types on the traffic level. AASHTO specification [36] classified the traffic loading into four classes (S is standard traffic (≤10 million ESALs), H is heavy traffic (10–30 million ESALs), V is very heavy traffic (≤30 million ESALs), and E is extremely heavy traffic (≥30 million ESALs) with standing traffic), depending on the J${}_{nr}$ values at high stress (3.2 kPa).

The neat and modified binders were tested using the MSCR test at different temperatures and high stress (3.2 kPa). The research team prepared two samples for each temperature (108 samples total), and the average value was used to determine the test result. Temperatures in the test varied from 46 to 70 ${}^{\circ }$C, with a 3 ${}^{\circ }$C increment. The influences of geopolymer and SBS on the asphalt binder grading, failing temperature, and traffic level are summarised in Table 11. The results obtained show that the geopolymer has a significant impact on the asphalt binder grading, which is consistent with the findings from [24,25].

The 8%GF binder had a high PG grading, failing temperature, and traffic level compared to the neat and modified binders with 4%GF and 12%GF. It had higher PG grading and failing temperatures than the 2%SBS binder did, and a similar traffic level. Modifying the asphalt binder with a combination of SBS and geopolymer produced the best results when compared to the other modifiers, confirming previous observations in G${}^{*}$/sinδ, J${}_{nr}$, and R. Table 11 offers guidance to asphalt pavement designers to select suitable modifiers considering the local temperature and traffic volume.

### 3.3. Hamburg Wheel Rut Test Results Analysis

**Figure 12:**
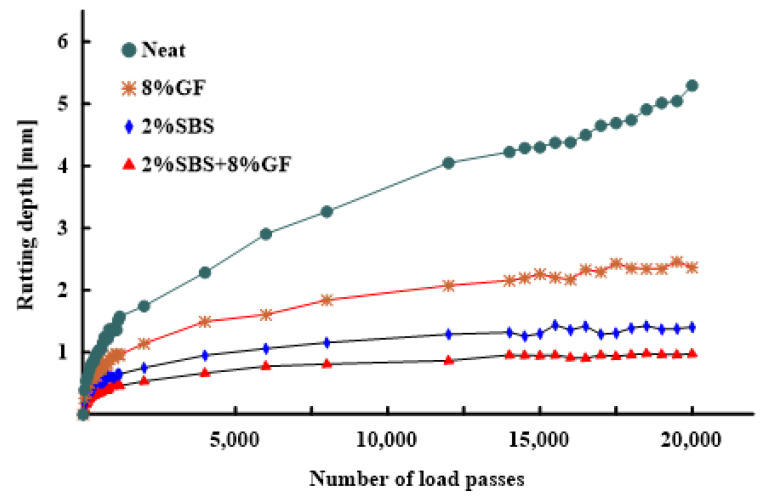
Additives effects on the rut depth of asphalt mixture.

Figure 12 shows the average rutting depth at different load passes for neat and modified asphalt binders. The findings of the HWRT tests describe the impacts of rutting and moisture damage, whereby moisture damage begins after the creep-to-stripping inflection point. The results showed that there is no negative effect of these additives on the moisture damage resistance. This finding indicates that geopolymer-modified asphalt binders have significant resistance to moisture damage, as also concluded by Rosyidi et al. [45]. As a result, the HMA with various additives had high moisture resistance. The hybrid binder achieved the highest resistance to rutting. The 2%SBS binder achieved rut resistance higher than the 8%GF binder. Compared to the neat binder, the permanent deformation decreased by 82%, 74%, and 55% by adding 2%SBS + 8%GF, 2%SBS, and 8%GF, respectively. Therefore, their abilities to resist permanent deformation are ranked as: 2%SBS + 8%GF > 2%SBS > 8% GF > neat, which confirms the earlier observation trends for MSCR test results in Figure 8, Figure 9, and Figure 11. These observations indicate that there is a good relationship between MSCR test results and asphalt mixture rutting, as concluded by various studies [14,46,47].

## 4. Conclusions

Using geopolymers as modifiers for asphalt binder proved to be an efficient solution to enhance rutting performance. Moreover, the paper’s findings offer guidance to asphalt pavement designers on selecting suitable modifiers considering the local temperature, stresses, and traffic volume. The following conclusions have been drawn:

- The shear complex modulus increased from 22 to 72 kPa, and the phase angle decreased from 74.5 to 65${}^{\circ }$ by adding 2%SBS + 8%GF at 46 ${}^{\circ }$C, showing that the viscoelastic behaviour becomes more elastic.
- G${}^{*}$/sinδ value of the hybrid binder was the highest among all the tested binders. This value reached 300% one of that of the neat binder. The combination of SBS and geopolymer appeared to have the highest rutting resistance.
- Geopolymer has a significant impact on the binder’s sensitivity to temperature, whereby the temperature sensitivity of G${}^{\prime }$ and G${}^{\prime \prime }$ for both unaged and RTFO modified asphalt binders decreased.
- The 2%SBS binder exhibited the highest creep recovery at low-stress levels, and the hybrid binder exhibited the highest creep recovery at high-stress levels, at all test temperatures.
- It was noted that the stress levels, temperature, and polymer type had important effects on the accumulated strain, whereby the modified asphalt binders maintained the lowest creep strains. The hybrid binder showed the lowest accumulated strain of the modified binders.
- The MSCR test results indicated that adding a geopolymer to SBS can enhance binder’s ability to withstand extreme (E) and very heavy (V) traffic under high stress and temperature. Therefore, the combination of geopolymer and SBS could be used to improve the rutting resistance capabilities of asphalt binders in hot countries.
- Adding 8%GF to the neat binder enhanced the rutting resistance of the asphalt mixture, which reduced the rut depth by 55%. The combination of the SBS and GF (2%SBS + 8%GF) reduced the rut depth to 82%.
- The MSCR test results could be used to develop preliminary indications of the permanent deformation of the asphalt mixture, as the results were aligned with the HWRT results.

In this study, the idea of using fly ash as an aluminosilicate source during the preparation of geopolymer and then utilising it as a modifier for asphalt binder provides a practical explanation for improving asphalt pavement rut resistance, eliminating the threat of environmental disposal of fly ash, and reducing CO${}_{2}$ emissions and fuel consumption due to asphalt binder extraction and transportation. Since a few investigations have studied the rheological and mechanical performances of geopolymer-modified asphalt binder and mixtures, there remains a lack of evidence on the laboratory evaluation of geopolymer-modified asphalt binders and mixtures. The effects of fly-ash-based geopolymer content on the fatigue and low-temperature crack resistance of asphalt binder and mixture have not been investigated yet. Additionally, the influences of ageing and climate change conditions on the geopolymer-modified asphalt binder and mixture should be evaluated. Therefore, it is recommended to investigate the fatigue and low-temperature performances of geopolymer-modified asphalt binders and mixtures using static and dynamic tests.

Globally, SBS was widely employed in many nations, and it had a considerable impact on the rheological performance of the asphalt binder. As a result, comparing the promised effects of employing the geopolymer as a modifier with the results of another modifier, such as SBS, could motivate the use of the geopolymer during pavement construction. Another interesting finding is that the combination of geopolymer and SBS led to a promising change in the viscoelastic behaviour of the asphalt binder, increasing the storage modulus (elastic behaviour) and loss modulus (viscous behaviour). These changes indicate the need for using different combinations with different percentages of geopolymer and SBS, and investigating the effects of various factors, such as temperature, curing time, and mixing procedure, on the behaviour of the asphalt binder; and then the effects of these factors on the polymer structure should be discussed. Therefore, additional physical, chemical, and microstructural investigations are recommended.

## Figures and Tables

**Table 1:** Fly ash chemical composition (test results obtained from materials supplier).

Constituent (%)	SiO${}_{2}$	Al${}_{2}$O${}_{3}$	Fe${}_{2}$O${}_{3}$	CaO	MgO	SO${}_{3}$	Na${}_{2}$O	MC	LOI
**Fly ash**	57.2%	23.5%	3.8%	9.3%	1.0%	0.2%	2.43%	0.06%	0.77%

Note: MC is Moisture Content and LOI is Loss on Ignition.

**Table 2:** Geopolymer and SBS percentages in the modified asphalt binders.

Parameter	Neat (%)	4%GF (%)	8%GF (%)	12%GF (%)	2%SBS (%)	Hybrid (2%SBS + 8%GF) (%)
**Asphalt**	100	96	92	88	98	90
**Geopolymer**	0	4	8	12	0	8
**SBS**	0	0	0	0	2	2
**Total**	100	100	100	100	100	100

**Table 3:** Aggregate size distribution.

Sieve Size (mm)	Passing (%)	Control Point (Maximum)	Control Point (Minimum)
19	100.0		
12.5	95.0	100	90
9.5	83.0	90	28
4.75	58.0		
2.36	40.0	58	28
1.18	19.0		
0.6	12.0		
0.3	8.0		
0.15	4.5		
0.075	3.0	10	2

**Table 4:** Two-way ANOVA testing of the viscosity of neat and modified asphalt binders.

Source of Variation	SS	df	MS	F	*p*-Value	F-Crit
**Binder Modification**	7,859,537	6	1,309,923	284,324.4	1.9 × 10${}^{-151}$	2.23
**Temperature**	7,385,222	1	7,385,222	1,602,994	2.4 × 10${}^{-154}$	3.98
**Interaction**	2,701,719	6	450,286.4	97,736.59	3.3 × 10${}^{-135}$	2.23
**Within**	322.5	70	4.607143			
**Total**	17,946,801	83				

**Table 5:** F-test comparing shear modulus of modified binders to the neat binder.

Binder	*p*-Value	Significance	Percentage Increase in G${}^{*}$
4%GF	0.06	Insignificant	65%
8%GF	0.01	Significant	107%
12%GF	0.05	Insignificant	73%
2%SBS	0.26	Insignificant	31%
2%SBS + 8%GF	4.8 × 10${}^{-4}$	Strongly significant	222%

**Table 6:** Geopolymer’s effect on the regression slope of storage and loss modulus.

Binder	Slope ∣G${}^{\prime }$∣	Slope ∣G${}^{\prime }$∣ RTFO	Slope ∣G${}^{\prime \prime }$∣	Slope ∣G${}^{\prime \prime }$∣ RTFO
Neat	9.114	8.864	6.919	7.029
4%GF	8.854	8.359	6.856	6.809
8%GF	8.582	8.233	6.800	6.802
12%GF	8.742	8.330	6.816	6.840
2%SBS	6.792	6.975	6.172	6.317
Hybrid	7.869	7.246	6.517	6.401

**Table 7:** Temperature’s effect on the rutting factors of neat and modified asphalt binders at 10 rad/s.

Binder		G${}^{*}$/sinδ			Modified/Neat	
	52 ${}^{\circ }$C	58 ${}^{\circ }$C	64 ${}^{\circ }$C	52 ${}^{\circ }$C	58 ${}^{\circ }$C	64 ${}^{\circ }$C
Neat	9.73	4.38	2.05	1.00	1.00	1.00
4%GF	16.82	7.58	3.56	1.73	1.73	1.74
8%GF	21.52	9.81	4.63	2.21	2.24	2.26
12%GF	17.81	8.08	3.81	1.83	1.85	1.86
2%SBS	14.68	7.17	3.64	1.51	1.64	1.78
Hybrid	36.71	17.51	8.63	3.77	4.00	4.21

**Table 8:** Two-way ANOVA testing of the rutting parameters versus temperature.

Source of Variation	SS	df	MS	F	*p*-Value	F-Crit
**Binder Modification**	10,897.368	5	2179.474	2213.829	9.88 × 10${}^{-44}$	2.477
**Temperature**	88,602.255	5	17,720.45	17,999.78	4.33 × 10${}^{-60}$	2.477
**Interaction**	13,562.988	25	542.52	551.07	1.65 × 10${}^{-39}$	1.815
**Within**	35.441	36	0.985			
**Total**	113,098.052	71				

**Table 9:** Frequency effects on the rutting factors of neat and modified asphalt binders.

Binder	∣G${}^{*}$∣/sinδ	∣G${}^{*}$∣/sinδ	Modified/Neat	Modified/Neat
	10${}^{-2}$ H${}_{z}$	10 H${}_{z}$	10${}^{-2}$ H${}_{z}$	10 H${}_{z}$
Neat	0.07	45.71	1.00	1.00
4%GF	0.20	73.99	2.84	1.62
8%GF	0.27	91.67	3.86	2.01
12%GF	0.22	78.14	3.14	1.71
2%SBS	0.27	59.62	3.86	1.3
Hybrid	0.62	140.12	8.86	3.07

**Table 10:** Two-way ANOVA testing of the rutting parameters versus frequency.

Source of Variation	SS	df	MS	F	*p*-Value	F-Crit
**Binder Modification**	5423.876	5	1084.775	892.184	5.45 × 10${}^{-15}$	3.106
**Frequency**	39,625.466	1	39,625.466	32,590.36	5.61 × 10${}^{-22}$	4.747
**Interaction**	5309.938	5	1061.988	873.442	6.19 × 10${}^{-15}$	3.106
**Within**	14.590	12	1.216			
**Total**	503,73.871	23				

**Table 11:** Grading and traffic-level results of geopolymer, SBS, and hybrid asphalt binders.

Binder	PG	Temp. Range	Temp. Range	Temp. Range	Temp. Range
		for S (${}^{\circ }$C)	for H (${}^{\circ }$C)	for V (${}^{\circ }$C)	for E (${}^{\circ }$C)
Neat	58	≥57.0	57.0–53.0	53.0–49.0	≤49.0
4%GF	64	≥62.0	62.0–57.0	57.0–53.0	≤53.0
8%GF	70	≥64.0	64.0–59.0	59.0–55.0	≤55.0
12%GF	64	≥62.5	62.5–57.0	57.0–53.0	≤53.0
2%SBS	64	≥64.5	64.5–59.5	59.5–55.0	≤55.0
Hybrid	76	≥70.0	66.0–70.0	61.0–66.0	≤61.0

## Data Availability

All data used during the study appear in the submitted article.
